# Supplementary material for: Dendritic Organic Electrochemical Transistors Grown by Electropolymerization for 3D Neuromorphic Engineering
Source: Adv Sci (Weinh). 2021 Oct 29;8(24):2102973. doi: 10.1002/advs.202102973 (PMC8693061; doi:10.1002/advs.202102973)
Supplement: Supplementary file 1 — Supporting Information [file ADVS-8-2102973-s001.pdf]

WILEY-VCH

© 2021 Wiley-VCH GmbH

ADVANCED SCIENCE
Open Access

Supporting Information

for *Adv. Sci.*, DOI: 10.1002/advs.202102973

Dendritic organic electrochemical transistors grown by electropolymerization for 3D neuromorphic Engineering

Kamila Janzakova, Mahdi Ghazal, Ankush Kumar, Yannick Coffinier, Sébastien Pecqueur, and Fabien Alibart**

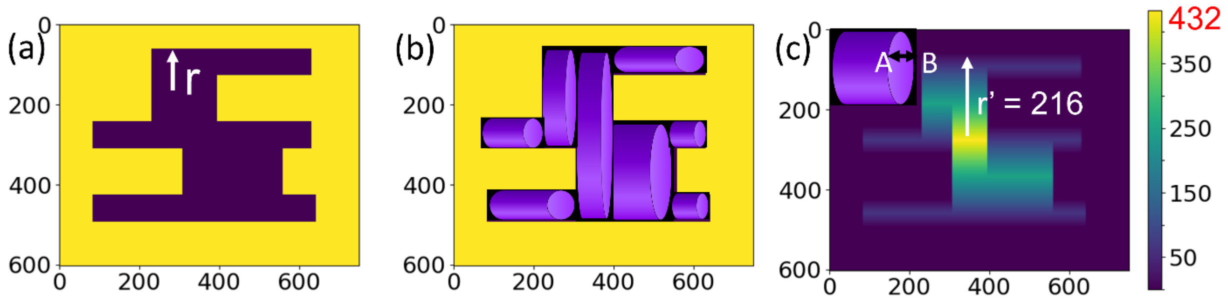

Figure S1: (a) An example of a 2d image, (b) reconstructed 3d image based on cylindrical shapes, (c) 3d profile of the image.

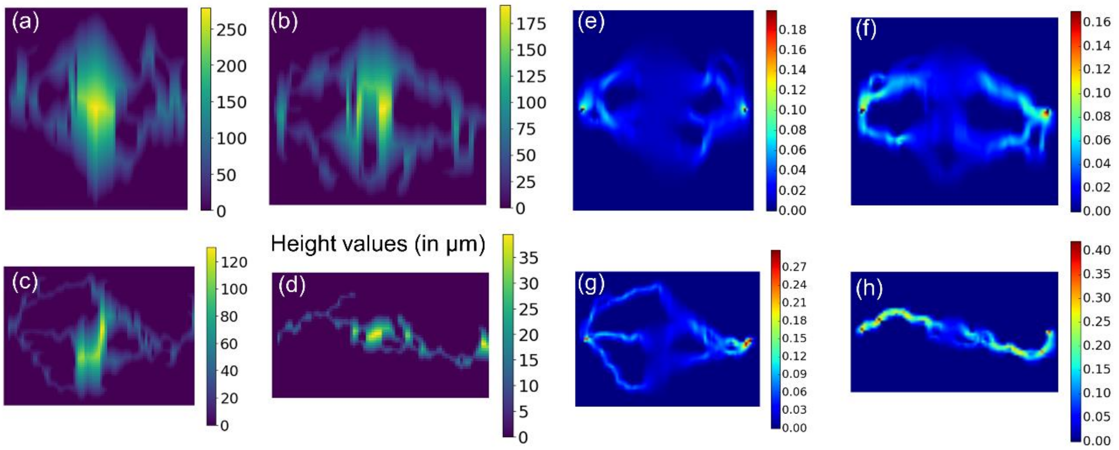

Figure S2: (a-d) 3-d morphology of dendrites based on multiple cylinder assumption and corresponding (e-h) normalized current density map for the dendrite obtained at fp = 40-320 Hz based on image analysis and electrical simulations.

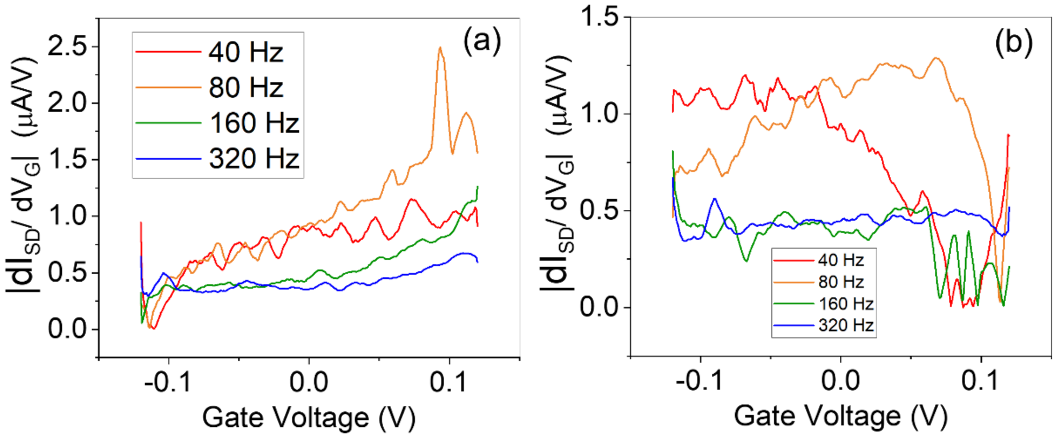

Figure S3: Variation in the derivative of source-drain current with the applied gate voltage during the (a) increasing and (b) decreasing values of the gate voltage.

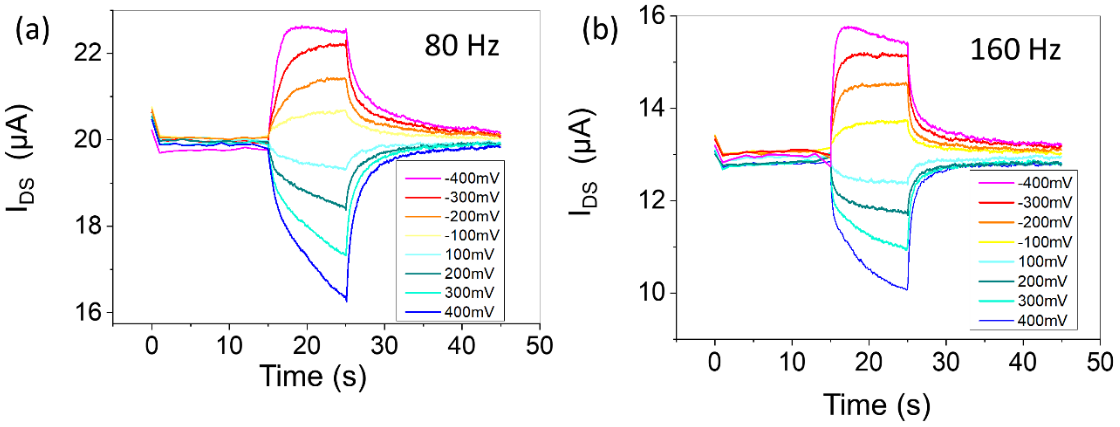

Figure S4: Source-Drain current responses for dendritic OECTs grown at (a) fp = 80Hz and (d) fp = 160 Hz with pulse amplitude from -0.4 to 0.4 V.

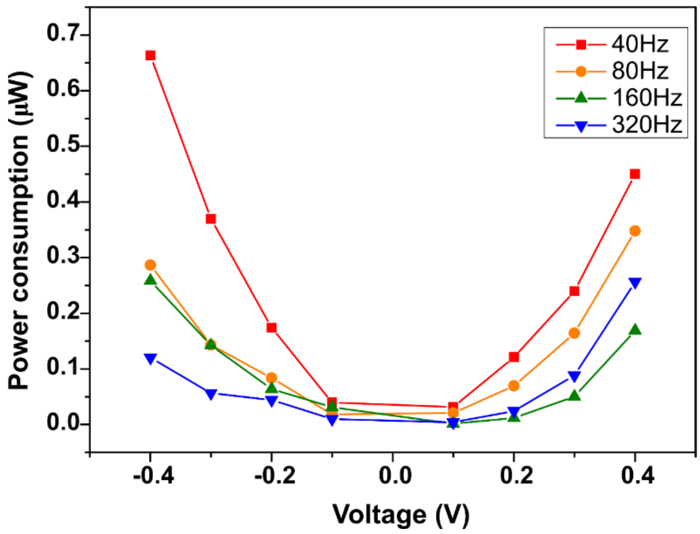

Figure S5: Power-consumption of dendritic OECTs grown at 40 Hz, 80 Hz, 160 Hz, 320 Hz at Vg programming pulses from -0.4V to 0.4V. Power consumption was calculated by measuring the gate current during programming.
